# Evolution of Peripheral Visual System in the Apoidea: A Role for Food Item Mobility?

**DOI:** 10.1002/ece3.73608

**Published:** 2026-05-05

**Authors:** Chiara Francesca Trisoglio, Andrea Ferrari, Matteo Brilli, Michele Zilioli, Carlo Polidori

**Affiliations:** ^1^ Department of Environmental Science and Policy (ESP) University of Milan Milan Italy; ^2^ Department of Biosciences University of Milan Milan Italy; ^3^ Museo Civico di Storia Naturale Milan Italy

**Keywords:** bee, eye size, foraging, ocelli size, prey, wasp

## Abstract

The morphology of insect visual system was often linked with resource type and resource‐finding strategies. Since larger compound eyes and ocelli altogether improve image resolution, capturing light and motion perception, one may expect that insects specialised in chasing very mobile resources possess such morphological optimisation. We tested this hypothesis in females of Apoidea (Hymenoptera), which include bees (which just land on flowers to collect pollen), wasps hunting for weakly mobile prey that are not chased in flight (e.g., beetles, aphids) (*LM*r‐species) and wasps which prey upon highly‐mobile flying prey (e.g., flies, bees) (*HM*r‐species). By analysing 77 species, we have found that, once taken into account the head width, *HM*r‐ and *LM*r‐wasps did not differ in eye size, but had larger eyes than bees. Median ocellus diameter, on the other hand, did not differ between bees and wasps, though *HM*r‐wasps had larger ocelli than *LM*r‐wasps. The observed variations were largely dependent on head width, with the eye enlargement being faster in wasps than in bees as the head size increased. Phylogenetically‐corrected models highlighted a strong effect of common ancestry on morphological variation but confirmed the significant effect of food mobility for the relative eye size. Ancestral state reconstructions suggested a shift to relatively smaller eyes in correspondence with the shift to pollen provisioning, while things seemed to be more complex with the relative ocellar size, likely because of the negative allometry of this trait, especially strong in bees. We may conclude that the need to hunt rapidly moving prey in flight has likely contributed to the evolution of the visual system in Apoidea.

## Graphical abstract

Since larger compound eyes and ocelli altogether improve vision, one may expect that insects specialised in chasing very mobile resources possess such morphological optimisation. By analysing 77 species of bees and wasps, we have found that wasps had larger eyes, but not larger ocelli, than bees. The observed variations were also dependent on head width and on common ancestry. Ancestral state reconstructions suggested a shift to relatively smaller eyes in correspondence with the shift to pollen provisioning.

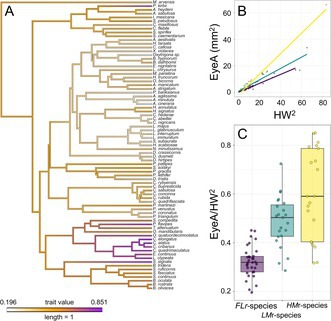

## 1 Introduction

The visual system of most flying insects is composed of two functional structures, a pair of compound eyes and generally three dorsal ocelli (Land and Nilsson 2012). Apposition compound eyes are comprised of several hundreds or thousands of visual units called ommatidia, optically isolated from each other. Each ommatidium usually contains a fixed number of neuronal photoreceptors, pigment cells and lens‐secreting cone cells (M. F. Land 1998). This design allows for precise image recognition, fast motion detection and light capture (Klowden and Palli 2023). The size of the compound eye affects these properties and ultimately determines the image resolution (acuity) (M. F. Land 1997; Jander and Jander 2002).

The dorsal ocelli consist of simple lens eyes located on the dorsal region of the head (Baird and Yilmaz 2023). Compared to compound eyes, ocelli have higher processing speeds, higher sensitivity to visible light, and to mono‐ or dichromatic wavelengths (UV and green). They help regulate the timing of activities or set the circadian system (Eaton et al. 1983; Rence et al. 1988), aid in flight stabilisation (Taylor 1981; Stange 1981; Parsons et al. 2010), and mediate orientation using polarised light (Fent and Wehner 1985; Taylor et al. 2016). By being highly sensitive to light, ocelli essentially seem to be horizon detectors that help make a fast correction during flight. However, differently to compound eyes, ocelli, except for a few observed cases, are under‐focused and not able to form images (Goodman 1981; Honkanen et al. 2018).

The processing of visual information is involved in a variety of insect behaviours, such as triggering escape, chasing mates, and pursuing prey (Buschbeck and Friedrich 2008; Land and Nilsson 2012; Srinivasan and Zhang 2000). Thus, it is not surprising that different studies highlighted that both compound eyes and ocelli are under strong selective pressures, so that variation in their morphology is often linked with variation in life history or behavioural traits (Greenfield 2002; Dötterl and Vereecken 2010). In particular, the visual system is essential for resource‐finding activities and therefore has evolved to optimize resource acquisition (Polidori et al. 2020; Kelber et al. 2006). For example, nocturnal or crepuscular bees, wasps and ants have enlarged compound eyes and ocelli to adequately navigate under low light conditions while foraging (Liporoni et al. 2020; Wcislo et al. 2004; Warrant et al. 2006; Johnson and Rutowski 2022). In species of *Myrmecia* ants, the mode of locomotion (reproductive alates vs. workers) and time of activity (diurnal vs. nocturnal) influence the ocellar structure of similar‐sized individuals (Narendra and Ribi 2017). Similarly, carabid beetles which are diurnal hunters have larger eyes than nocturnal species (reviewed in Meyer‐Rochow and Lindström 2025). Prompt detection of mating partners drove the morphology of the visual system in bees, with territorial males often having larger eyes compared to non‐territorial males (Leys and Hogendoorn 2008). Kleptoparasitic fly species (Diptera: Miltogramminae), which engage in complex host‐trailing flights (satellite flights) to find host nests, possess larger compound eyes and ocelli than closely related species that find the host nests by inspecting the soil surface (Polidori et al. 2022). Finally, dragonflies are formidable aerial predators and are well known for their exceedingly enlarged compound eyes suited to detect and capture rapidly moving prey (Combes et al. 2012; Olberg 2012).

Regardless of resource‐finding strategies, the insect visual system is also influenced by body size, with several studies having found a positive correlation between body size and the size of compound eyes and ocelli. However, these relationships can be allometric, with larger individuals, within a species, having relatively smaller structures (Jander and Jander 2002; Araújo et al. 2023; Kapustjanskij et al. 2007).

Here, we investigated the evolution of the visual system in the Apoidea, a large lineage of aculeate Hymenoptera characterised by an important variation in life‐history traits, including larval diet (Danforth et al. 2019; O'Neill 2001). Currently, the Aculeata includes one superfamily of bees and wasps (Apoidea), six other superfamilies of wasps (Vespoidea, Pompiloidea, Chrysidoidea, Sierolomorphoidea, Tiphioidea, Thynnoidea, and Scolioidea), and one superfamily of ants (Formicoidea) (Branstetter et al. 2017). The Apoidea, which encompass all bees (7 families) and a large group of predatory wasps (11 families) (Sann et al. 2021), include lineages which specialised in different food resources provided by adults to the developing brood. Bees collect pollen (Polidori et al. 2025), while wasps hunt for a variety of arthropods, with each species highly specialised in their prey range (from few families within a single order to a single species) (Bohart and Menke 1976; Evans and O'Neill 2007). Both bees and wasps are frequent flower visitors because adult wasps feed on nectar, as well as hunting prey to feed the larvae.

The collection of these different resources may require different visual needs. From one side, bees land on flowers to collect pollen, a static, immobile resource (Polidori et al. 2025). On the other side, wasps catch mobile items for provisioning. Some wasp species are specialised in hunting mobile prey such as grasshoppers and beetles which are, however, not captured in flight but typically on plants (Casiraghi et al. 2001; Polidori et al. 2010; Santoro et al. 2011). Conversely, other wasp species hunt fast‐flying bees or flies (Ballesteros et al. 2016; Polidori et al. 2009). These fast‐flying insects are also highly elusive, leading to higher generalization in wasp species hunting for such prey (Polidori et al. 2013). Foraging specialization for both ‘prey mobility’ types evolved within different wasp families (Bohart and Menke 1976) and even within a few genera (Polidori 2011; Wurdack et al. 2017), making Apoidea a good model group for comparative studies on morphological traits linked with foraging behaviour.

Because tracking and chasing a fast, highly mobile target may require better visual acuity, light motion detection and flight stabilisation, we then here hypothesised that wasp species targeting highly mobile prey possess larger compound eyes and ocelli, compared with wasps hunting for more slowly‐flying or non‐flying prey, and that wasps possess larger vision‐related structures compared with bees. We particularly predicted that (1) compound eyes and ocelli size changed in wasps in correspondence of shifts in ‘prey mobility’ specialization, and (2) since bees derived from apoid wasps (Sann et al. 2021), compound eyes and ocelli shrank in correspondence with shifting to pollen diet, since bees were released from the pressure of hunting for mobile items.

## 2 Methods

### 2.1 Study Species

The dataset is composed of measurements made on a total of 265 females (2–5 per species) spanning 77 species of Apoidea, mostly from the Palearctic region. Following the most recent classification of Sann et al. (2021), the species set spans 12 out of the 18 families of Apoidea (Dataset S1). We used only females because we specifically aimed to analyse possible evolutionary pressure of prey hunting on the visual system, and males do not hunt. According to their ‘food item mobility’, we categorised each species as either foraging for ‘flower’ pollen (*FLr*‐species), for ‘low mobility’ prey (*LMr*‐species), or for ‘high mobility’ prey (*HMr*‐species). For wasp species, we inspected the known prey spectrum by checking relevant literature for categorisation (Bohart and Menke 1976; O'Neill 2001; Evans and O'Neill 2007; Polidori 2011, 2013). Spiders, beetles, grasshoppers, mantises, aphids and lepidopteran larvae were ranked as ‘low mobility’ prey; while bees, flies and adult butterflies were ranked as ‘high mobility’ prey.

The studied specimens were obtained from field sampling activities by the authors and collaborators (private collections), from academic museums (Colección de Entomología de la Universidad Complutense de Madrid, Museo di Zoologia Università degli Studi di Milano), and from public museums (Museo Civico di Storia Naturale di Milano, Museo de Ciencias Naturales of Madrid) (Dataset S1). Depending on the sample (i.e., the facilities at the different labs where the study was carried out), pictures of the head in frontal position were taken either under a stereomicroscope (with a Canon PowerShot S50 digital camera mounted on a Leica MS5 stereomicroscope at the Museo Civico di Storia Naturale di Milano, with a LEICA DFC7000 T camera mounted on a Leica M205 FCA stereomicroscope at the University of Milan, or with a Leica DFC450 digital camera mounted on a Leica M165C stereomicroscope at the Museo de Ciencias Naturales of Madrid); or in a Scanning Electron Microscope (SEM) (JEOL JSM 5610 LV (JEOL Ltd., Tokyo, Japan) at the Museo Civico di Storia Naturale di Milano and at University of Milan or with an ESEM QUANTA 200 microscope (FEI Company, Oregon‐USA) at the Museo Nacional de Ciencias Naturales of Madrid).

### 2.2 Morphological Traits

**FIGURE 1:**
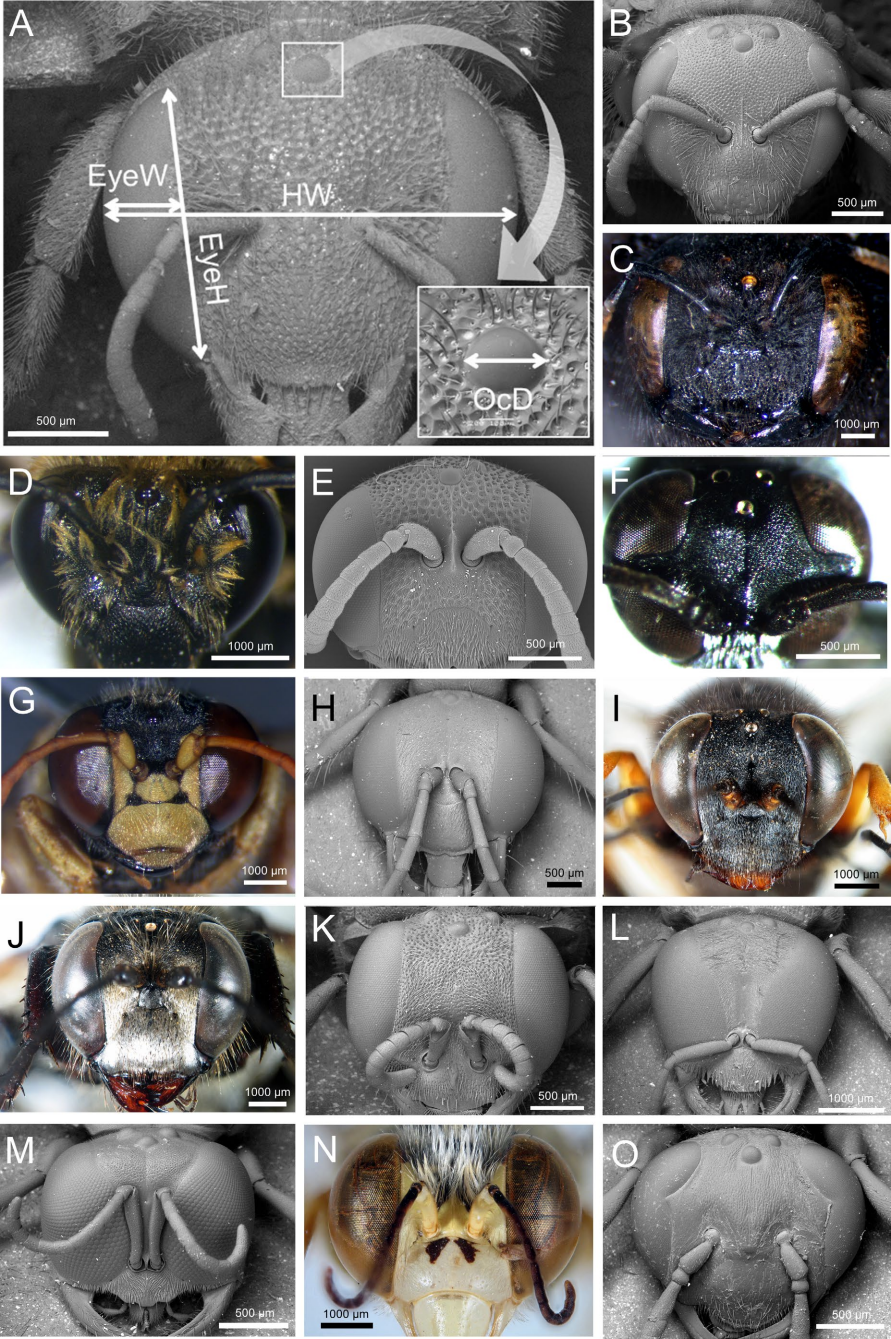
Examples of pictures of the studied species, taken either with SEM or with stereomicroscope. (A) *Anthidiellum strigatum* (with highlighted the measurements taken: HW: Head width, EyeW: Eye width, EyeH: Eye height, OcD: Ocellar diameter); (B) *Lasioglossum interruptum* ; (C) *Xylocopa violacea* ; (D) *Colletes hederae* ; (E) *Cerceris rubida* ; (F) *Trypoxylon attenuatum* ; (G) *Stizus continuus* ; (H) *Ammophila heydeni* ; (I) *Sceliphron spirifex* ; (J) *Sphex paludosus* ; (K) *Oxybelus mandibularis* ; (L) *Crabro alatus* ; (M) *Crossocerus elongatus*; (N) *Bembix olivacea* ; (O) *Philanthus venustus* . (A–D) *FLr*‐species; (E–J) *LMr*‐species; (K–O) *HMr*‐species.

On each specimen, we took four different measures (Figure 1A). The head width (HW), as the maximum distance between the outer margins of the eyes, was used in the analysis because a larger head could also harbour larger eyes and ocelli (e.g., Ferrari et al. 2024; Kerfoot 1967). On the compound eyes (hereafter, eye(s) for simplicity) (either left or right eye was randomly chosen) we measured two linear metrics in anterior view (Figure 1) (e.g., Polidori et al. 2022; Ferrari and Polidori 2024): the eye width (EyeW) (taken perpendicular to the sagittal plane of the body) and the eye height (EyeH) (taken parallel to the sagittal plane of the body). Then, we used these two measures to estimate the frontally visible eye area as a semi‐elliptic shape (EyeA = (*π* × [EyeW × EyeH])/2). We used the median ocellus (hereafter, ocellus for simplicity) diameter (taken parallel to the transversal body axis, in frontal view, OcD) as a proxy of its size (e.g., Ferrari et al. 2024). Ocelli were not measured in three species of the genus *Bembix* and one species of the genus *Stictia*, since in this tribe of Bembecidae (Bembicini) they are not fully developed and often substituted by mere arcuate grooves which are apparently not functional (Evans and O'Neill 2007). All the measurements were taken in ImageJ (Schneider et al. 2012) and are available in Dataset S1.

### 2.3 Phylogenetic Reconstruction

As a starting point for our phylogenetic reconstruction, we used alignments from Almeida et al. (2023) for the following genes: cytochrome oxidase I, RNA Polymerase II, NaK ATPase, wingless, LWRh, EF1a, CAD, 18S and 28S. Sequences for the species added for this work were retrieved from the NCBI nucleotide database searching by taxonomic name and then using blast to retrieve the above proteins; otherwise, we used blast on genome and transcriptome assemblies also downloaded from the NCBI database (Tables S1 and S2 for a full list of genomes and transcriptomes used). A summary of the alignments is shown in Table S3. We used sequences associated with the same species composing our dataset, except some cases in which analysed species were not sequenced and hence we used alternative, closely related species with sequences available (e.g., Polidori et al. 2013) (see Dataset S1).

Since for some of the species to be included in the tree only a subset of the orthologous sequences was available, we opted for a supertree approach using IQ‐TREE (Minh et al. 2020). To this aim, we implemented a partitioned analysis (Chernomor et al. 2016) where several alignments, each containing a subset of the species, were treated independently for then being integrated in a unique final phylogenetic tree. Model selection was therefore performed independently on each multi‐alignment, enabling us to select the best evolutionary model and to estimate its parameters for each of them. The analyses run were ModelFinder (Kalyaanamoorthy et al. 2017), followed by Maximum Likelihood tree reconstruction with 5000 ultrafast bootstrap replicates (Hoang et al. 2018).

### 2.4 Statistical Analysis

All the statistical analyses were run in R *v.4.4.2* (R Core Team 2024) and the plots were produced with the package *ggplot2* (Wickham 2016) using a colorblind‐friendly palette from *viridis* (Garnier et al. 2024). All the data are made available in Dataset S1.

Since some species were photographed under a stereomicroscope (lower resolution) and others in the SEM (higher resolution), we first verified that the two methods gave the same measures by using a Wilcoxon test for paired comparisons on subset individuals. We tested only ocellar diameter, which was the smallest measured structure. The two methods did not significantly differ (*W* = 65, *N* = 18, *p* = 0.37).

To test if the eye size and median ocellus diameter differed across food item mobility categories (*FLr*‐species, *LMr*‐species or *HMr*‐species) we run linear mixed models (using all individuals and species as a random factor) using *category*, *head width* (or head width^2^ in case of eye area) and their interaction as predictors. Then, to test if these variables increase differently with head size across food item mobility categories, pairwise comparisons of categories and comparisons of slopes between categories were tested using a Tukey post hoc test and ‘emtrends’ in the packages *car* (Fox and Weisberg 2019) and *emmeans* (Lenth and Piaskowski 2025). Simple linear regressions were used to test if the weighted size of eyes and ocelli (i.e., the measures divided by head width [ocellar diameter] or its quadratic value [eye area]) depended on the head width (or its quadratic value), that is to test if head size and visual trait size showed allometric relationships.

Then, to take into account shared ancestry (Martins and Hansen 1997), we used our phylogenetic tree (see above) to run Markov‐Chain Monte Carlo linear mixed models (MCMC‐LMM, with Gaussian family) (using all individuals) testing the effect of food item mobility on the weighted (i.e., relative to head size) visual traits, incorporating the phylogenetic tree as a random effect. These models were built in the *MCMCglmm* package (Hadfield 2010). Models were iterated for 3,000,000 times, the first 5000 iterations were discarded to enhance model convergence, and the sampling frequency was set to 1/500 iterations. The phylogenetic signal was estimated as phylogenetic heritability (*H* ^2^) since measures for all individuals, and not mean values *per* species, entered the models; this is equivalent to Pagel's ‘lambda’ (*λ*), which measures the strength of phylogenetic structure in the data when mean values per species are used (Pagel 1999), and approaches 1 when the signal is very strong.

Finally, the package *phytools* (Revell 2024) was used to reconstruct the ancestral states of food item mobility and weighted visual traits across the phylogenetic tree.

## 3 Results

**FIGURE 2:**
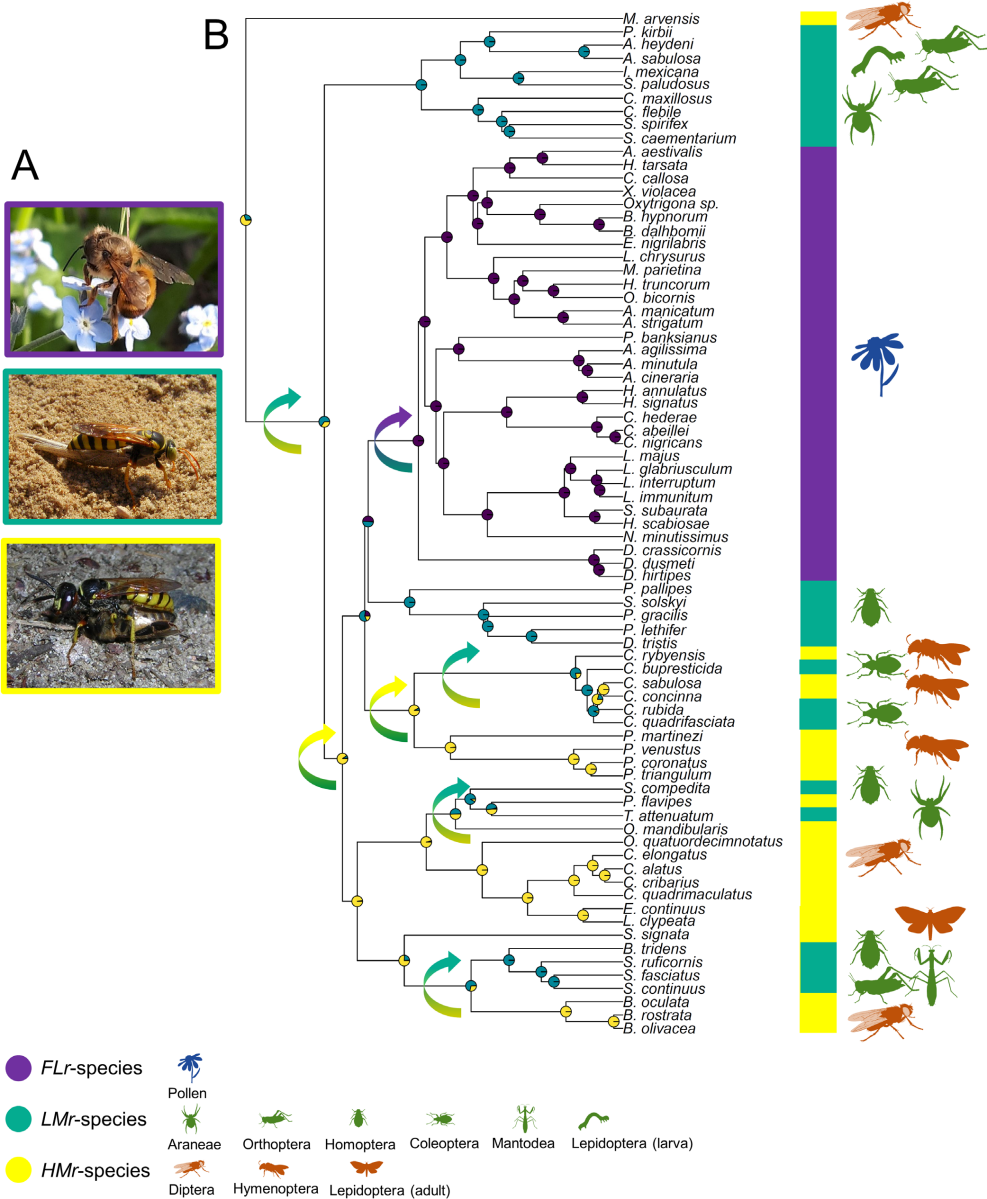
(A) Pictures of a *FLr*‐species ( *Osmia bicornis* ), a *LMr*‐species ( *Stizus continuus* ) and a *HMr*‐species ( *Philanthus triangulum* ); (B) Phylogeny of the studied species based on molecular data (see “Methods” section for details on the reconstruction), with ancestral state reconstruction of food item mobility. Coloured bars on the right of the tree report the food item mobility for each species. Prey hunted are depicted with small silhouettes (freely available from https://www.phylopic.org/) close to this bar. Curved arrows indicate suggested shifts (using the same colours of the states) between different food item mobility states.

In our set of species, the ancestral state reconstruction showed that hunting for highly mobile flying prey (Figure 2A) is the most likely ancestral foraging specialization in wasps and hence in the Apoidea as a whole (Figure 2B). From such behaviour, a shift to non‐flying or more slow‐flying prey (Figure 2A) likely occurred four times: at the rise of Sphecidae from ancestral Mellinidae, in the Bembicidae (ancestral prey: flies), in the Crabronidae (ancestral prey: flies) and once in the Philanthidae (ancestral prey: bees) (Figure 2B). Such weakly mobile prey (particularly aphids) persisted as the only type of prey in the Pemphredonidae, the sister group of bees in our sample (Figure 2B). From this wasp family, a single shift to pollen provision (Figure 2A) occurred when bees arose (Figure 2B). Few reversals from weakly mobile to highly mobile prey also potentially occurred: once at the rise of all wasps after separation from Sphecidae, and once at the rise of Philanthidae. Further reversals may have occurred (e.g., in the genus *Palarus*), but our phylogenetic reconstruction could not definitely confirm this (Figure 2B).

### 3.1 Compound Eye Size

**FIGURE 3:**
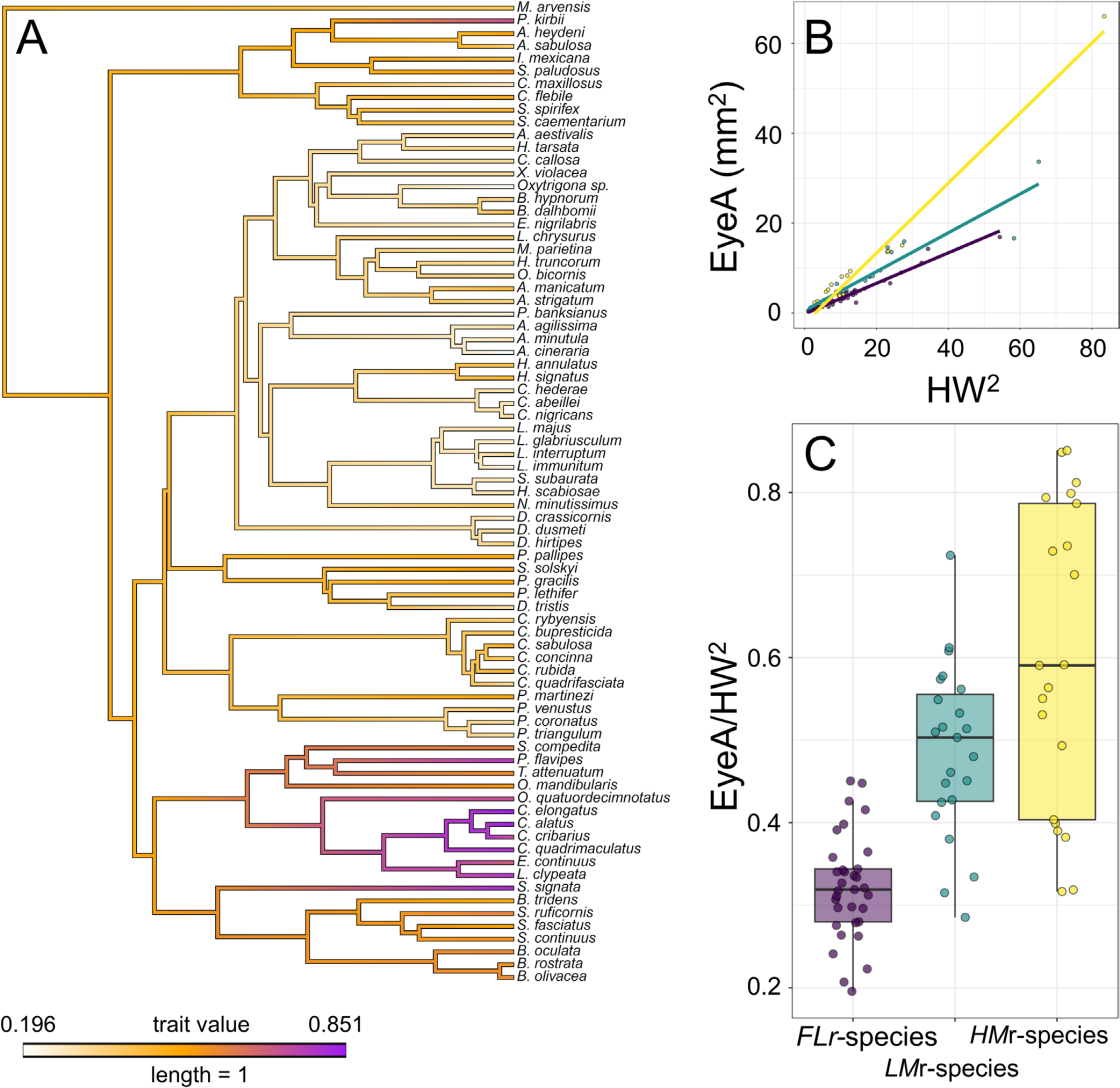
(A) Phylogeny of the studied species based on molecular data (see “Methods” section for details on the reconstruction), with ancestral state reconstruction of weighted eye area. (B) Relationship between squared head width and eye area across *FLr*‐species (violet), *LMr*‐species (green) and *HMr*‐species (yellow). Lines represent adjustment to linear models. (C) Box‐and‐Whiskers plots showing minimum, first quartile, median, third quartile, and maximum values of weighted eye area for *FLr*‐species (violet), *LMr*‐species (green) and *HMr*‐species (yellow). Each point represents a species.

The weighted eye size seemed to be ancestrally moderately high and maintained similar values across wasps, but notably increased further especially in Crabronidae and Bembicidae, hence in clades of *HMr*‐species, while clearly decreasing to lower values in bees (Figure 3A).

The linear mixed models revealed that squared head width accounted for the observed variation in eye area, with larger heads harbouring larger eyes (Table 1, Figure 3B). However, a statistically significant effect of food item mobility on eye area was also observed, with *FLr*‐species having smaller eyes than *HMr*‐species and almost statistically significantly when compared with *LMr‐species*, with no differences between the latter two groups (Table 2, Figure 3C).

**TABLE 1:** Results of the linear mixed models, showing the effect of food item mobility and head size (HW or HW^2^) on visual traits.

Trait	Predictors	*χ* ^2^	*p*
EyeA	Food item mobility	7.95	**0.046**
	HW^2^	202.01	**< 0.001**
	Food item mobility × HW^2^	14.89	**< 0.001**
OcD	Food item mobility	8.06	**0.018**
	HW	776.88	**< 0.001**
	Food item mobility × HW	3.32	0.19

*Note:* In bold the *p*‐value for significant results.

Abbreviations: EyeA, eye area; OcD, ocellar diameter.

**TABLE 2:** Results of the pairwise post hoc comparisons and slope comparisons among the three species groups, following the linear mixed models.

Tukey post hoc test					
Trait	Pairwise	Estimate	SE	*z‐*ratio	*p*
EyeA	*FLr* vs. *LMr*	−1.06	1.68	−0.628	0.080
	*FLr* vs. *HMr*	−4.78	1.72	−2.775	**0.015**
	*LMr* vs. *HMr*	−3.73	1.87	−1.990	0.11
OcD	*FLr* vs. *LMr*	0.01	0.008	1.88	0.14
	*FLr* vs. *HMr*	−0.01	0.009	−1.54	0.27
	*LMr* vs. *HMr*	0.03	0.009	2.99	**0.008**

Slope analysis					
Trait	Pairwise	Estimate	SE	*z‐*ratio	*p*
EyeA	*FLr* vs. *LMr*	−0.319	0.07	−4.44	**< 0.001**
	*FLr* vs. *HMr*	0.149	0.06	2.35	**0.048**
	*LMr* vs. *HMr*	0.467	0.07	6.26	**< 0.001**
OcD	*FLr* vs. *LMr*	0.01	0.008	1.88	0.14
	*FLr* vs. *HMr*	−0.01	0.009	−1.54	0.27
	*LMr* vs. *HMr*	0.03	0.009	2.99	**0.008**

*Note:* In bold the *p*‐value for significant results.

Abbreviations: EyeA, eye area; OcD, ocellar diameter.

The interaction between food item mobility and squared head width was significant, that is the strength of the effect of head area on eye area differed among species groups (Table 1). Indeed, while at similar head areas *FLr*‐species had smaller eyes than *LMr*‐species and *HMr*‐species, the difference in eye area among the three groups of species increased with head width (Figure 3B), and higher slopes were found in wasps than in bees and higher slopes in *HMr*‐species than *LMr*‐species (Table 2). Squared head width and weighted eye area showed no allometric relationships in either *FLr*‐, *LMr*‐ or *HMr*‐species (Table S4).

The phylogenetically‐corrected model revealed that *FLr*‐species had smaller eyes (relative to squared head width) than both *LMr*‐species and *HMr*‐species, and that *LMr*‐species had smaller eyes (relative to squared head width) than *HMr*‐species (Table 3). The estimated phylogenetic signal (*H* ^2^) was high (> 0.8), proving an important role of common ancestry on the observed variation in weighted eye area (Table 3).

**TABLE 3:** Results of the Markov‐Chain Monte Carlo linear mixed models, showing the effect of food item mobility on weighted size of visual traits (measured trait divided by HW for ocellar diameter and HW^2^ for eye area), after having taken into account the phylogenetic relationship between species.

Trait	*H* ^2^	Predictors	Post. Mean (95% CI)	*p*
Weighted EyeA	0.82	Food item mobility: *LMr*	0.31 (0.12–0.48)	**0.029**
		Food item mobility: *HMr*	0.22 (0.07–0.36)	**0.004**
Weighted OcD	0.79	Food item mobility: *LMr*	−0.0002 (−0.17–0.16)	0.99
		Food item mobility: *HMr*	0.006 (−0.01–0.02)	0.46

*Note:* CI: Lower and upper (respectively) 95% credible intervals. The baseline food item mobility (*FLr*) used in comparisons is not shown. In bold the *p*‐value for significant results. *H* ^2^, the phylogenetic signal.

Abbreviations: EyeA, eye area; OcD, ocellar diameter.

### 3.2 Ocellus Diameter

**FIGURE 4:**
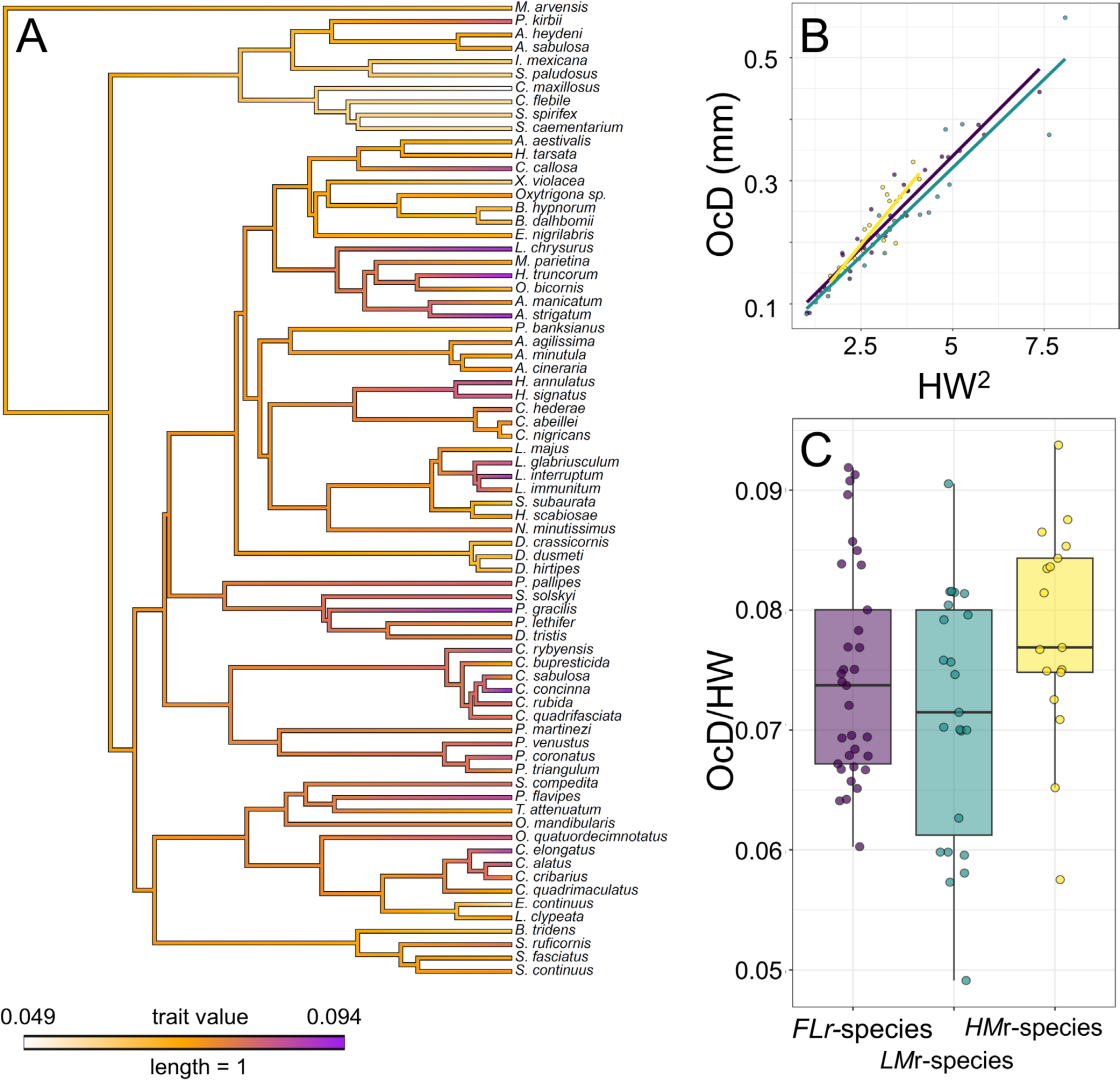
(A) Phylogeny of the studied species based on molecular data (see “Methods” section for details on the reconstruction), with ancestral state reconstruction of weighted median ocellar diameter. (B) Relationship between head width and ocellar diameter across *FLr*‐species (violet), *LMr*‐species (green) and *HMr*‐species (yellow). Lines represent adjustment to linear models. (C) Box‐and‐Whiskers plots showing minimum, first quartile, median, third quartile, and maximum values of relative ocellar diameter for *FLr*‐species (violet), *LMr*‐species (green) and *HMr*‐species (yellow). Each point represents a species.

The value of weighted ocellus diameter seemed to be ancestrally moderately high, then increased or decreased multiple times across the phylogeny (Figure 4A). For example, it generally increased in small bee species within Colletidae, Halictidae and Megachilidae. It also increased in various groups of wasps within Philanthidae (a mix of *LMr*‐ and *HMr*‐species), Crabronidae (essentially *HMr*‐species) and Pemphredonidae (*LMr*‐species) (Figure 4A). Ocellus seemed instead to shrink in a clade within Sphecidae (*LMr*‐species). Hence, shifts in ocellar size seemed to have occurred not according to predicted directions based on foraging specialization. However, overall, weighted median ocellus diameter seemed to increase more often than the opposite during apoid evolution (Figure 4A).

The linear mixed models revealed that head width accounted for the observed variation in ocellus diameter, with larger heads possessing larger ocelli (Table 1, Figure 4B). Ocellus diameter also significantly shifted according to food item mobility, but only because *LMr*‐species had smaller ocelli than *HMr*‐species (Tables 1 and 2, Figure 4C). The interaction between food item mobility and head width was not significant (Table 1), and the slopes did not differ between groups (Table 2, Figure 4B). Head width and weighted ocellus diameter showed a negative allometric relationship in *FLr* and *LMr*‐species, being especially strong in the former, with smaller species possessing disproportionately larger ocelli (Table S4).

The phylogenetically‐corrected model showed no difference in weighted ocellus diameter among *FLr*‐, *LMr*‐ and *HMr*‐species (Table 3). The estimated phylogenetic signal (*H* ^*2*^) was around 0.8, revealing an important role of common ancestry on the observed variation (Table 3).

## 4 Discussion

Among the possible drivers of visual system evolution in Hymenoptera, the temporal foraging window was by far the most tested, with extensive evidence that nocturnal or dim‐light bees, wasps and ants have larger eyes and larger ocelli than strictly day‐active related species (Warrant 2008; Warrant et al. 2006; Greiner et al. 2004, 2007; Greiner 2006; Moser et al. 2004). A lesser studied factor concerns the reproductive context, with species with territorial males having larger eyes compared to species with non‐territorial males (Leys and Hogendoorn 2008; Streinzer and Spaethe 2014). Food resource type and behaviours related with food access were, in contrast, not investigated to date. Albeit several general statements in the literature already expressed the idea of a link between visual system and prey type in wasps (e.g., Evans 1966; Bohart and Menke 1976), it was only formally verified here, showing that the size of eyes, but not the size of ocelli, likely shifted according to the food item mobility.

It is well known that the size of the retinal image increases with eye size, leading to an improved visual acuity for larger eyes (Cronin et al. 2014; Land and Nilsson 2012). In the context of predation, hunting for flying, highly mobile prey such as bees or flies may thus require larger eyes. Predation was often invoked as a relevant driver for eye evolution in animals, and the ability to detect and track prey is hypothesised as one of the earliest functions of the eyes (Cronin 2005). Our results support the role of predatory behaviour on eye adaptations, confirming our hypothesis on the link between food item mobility and eye morphology. Indeed, not only do bees have smaller weighted eyes than wasps, but also wasps hunting for highly mobile prey have larger weighted eyes than wasps hunting for weakly mobile prey. Such a difference is maintained after having corrected for common ancestry. Phylogenetic signal was however high, likely because *HMr*‐species are concentrated in few wasp lineages and because in the few lineages including both *HMr*‐ and *LMr*‐species, differences between the two groups were not strong.

A good example of this is the family Philanthidae, where bee‐hunters and beetle‐hunters show similar eye area. On the other hand, in Bembicidae, fly‐hunters had larger eyes than grasshopper‐hunters. This point is interesting, since specialization in brachyceran fly prey (the group of Diptera hunted by wasps) could have been especially important in shaping eye morphology. Flies are extremely fast‐flying insects able to quickly jump and take flight and to rapidly change direction in flight (Gnatzy and Tautz 2025). Perhaps hunting a fly is more complex not only compared with a weakly mobile prey, but also compared with a bee (Tinbergen 1935). The two main groups of fly‐hunters in our dataset are within Crabronidae and Bembicidae, and both have optimized morphology to catch flies, having prominent eyes that occupy much of the head, helping them to swift, hover, and turn rapidly while hunting (Iersel 1952).

The shift from predation to pollen collection was clearly associated with a reduction of relative eye size. Bees arose from a group of wasps exclusively hunting for weakly mobile prey. In our sample, the closest family to bees was the Pemphredonidae, which are also closely related to the aphid‐hunting Ammoplanidae, the recognized sister family to bees (Sann et al. 2021). Inspection of the literature suggests that also these small wasps, which are specialised in hunting thrips (almost static insects) on flowers, have small eyes relative to their head width (e.g., Carbonell Font 2025). This confirms a likely progressive reduction in these visual structures approaching the shift to pollen diet in bees.

Our findings on eye size agree with studies on other insect predators. For example, dragonflies (Odonata) are well known for their exceptional vision and skills to pursue and capture flying prey thanks to their very large compound eyes (Corbet 1999). More relevant for our study is the case of spiders (Araneae): for example species in the family Salticidae have large eyes, allowing them to stalk and pounce on their prey, while species in the family Thomisidae have small eyes because they hunt by hiding inside flowers to surprise and catch insects that land to feed (Uetz 1992; Chong et al. 2024). Another similar scenario to our case is that of miltogrammine flies (Diptera) which are kleptoparasites of bees and wasps. In this fly lineage, there are species in which females perform ‘satellite flight’, closely tracking their host in flight, while other species find their hosts by scanning host nest entrances (Piwczyński et al. 2017; Spofford and Kurczewski 1990). Polidori et al. (2022) showed that such behavioural divergence is associated with a difference in visual system, with ‘satellite’ species possessing larger eyes than the species from the latter group. This is very similar to what we found in our study: pursuing a fast‐flying item requires larger eyes compared with collecting for static (pollen) or weakly mobile/non‐flying items.

Certainly, large eyes evolved also in some insect groups which predate on non‐flying arthropods. For example, big‐eyed bugs in the genus *Geocoris* (Heteroptera) use their prominent eyes to find very small prey such as mites, aphids and insect eggs (Hagler and Cohen 1991), while praying mantises (Mantodea) take advantage of their large eyes to quickly stalk and ambush their prey (Corrette 1990). Hence, it is not surprising that the *LMr*‐species in our sample have larger eyes than bees. But hunting for very quick‐flying prey seemed to require even larger eyes.

In contrast to our hypothesis, there was not a clear association between food item mobility and median ocellus size. Ocelli are known to be tuned to capture light and hence to have a role in light metering, maintaining stability during flight, and motion perception (Warrant et al. 2006; Baird and Yilmaz 2023), which we suggested to be relevant aspects for a fast‐flying prey wasp. We have evidence from the literature that ocellar size is increased in nocturnal Hymenoptera (Warrant 2008; Warrant et al. 2006; Greiner et al. 2004, 2007; Greiner 2006; Moser et al. 2004) and in miltogrammine flies performing satellite flights (Polidori et al. 2020). Instead, in our study larger species had larger ocelli and—especially for bees—smaller species had disproportionately larger ocelli. Furthermore, wasps hunting for weakly mobile prey have smaller ocelli than those hunting for highly mobile prey, but this difference disappeared in phylogenetically corrected contrasts. We suggest that, at least partially, the found allometric relationships, which seem common for the insect visual system (Jander and Jander 2002; Perl and Niven 2016), are responsible for the lack of clear evolutionary association between ocellar size and foraging ecology. We cannot exclude that ocelli could be even less important for hunting than for finding flowers. As a matter of fact, two genera of fly‐hunters here studied, *Bembix* and *Stictia*, lack well‐formed and probably functional ocelli. Kerfoot (1967) showed that in bees, the greater the absolute size of the ocelli, the lower the illumination at which a given species forages. Perhaps very small bees have to maintain adequate size of the ocelli to properly forage, and this may lead to the observed negative allometry.

Some methodological limitations of our study (primarily the use of stereomicroscopy for many samples) did not allow to further investigate eye morphology by considering the number and size of ommatidia, and the inter‐ommatidia angle (i.e., the angle between the centres of two adjacent ommatidia), which altogether play a role in determining visual spatial resolution and light sensitivity (Snyder 1979; M. F. Land 1997; Jander and Jander 2002; Bartholomée et al. 2025). In addition, while the relationships among all bee families and most wasp families are consistent with the most recent reconstructions (Krichilsky et al. 2025), our phylogenetic reconstruction—Mellinidae + (Sphecidae + [Crabronidae + Bembicidae]—could not precisely match alternative and elsewhere supported evolutionary hypotheses: i.e., Mellinidae + [Sphecidae + Crabronidae] + Bembicidae). Though it remains important to elucidate this point, we believe that the found effects of food item mobility on visual system would be essentially the same.

Additionally, we could not use sequences for all the studied species, and hence branch lengths may not be that precise despite keeping the same topology in terms of relative distance among the studied species. Hence, caution is needed if the missing species has a highly unique niche not shared by its relatives. However, closely related species often exhibit similar traits, meaning that the substitution process often retains the ecological signal. Although rare, we specifically addressed this point by using proxy species with the same prey as the studied species (e.g., in the genus *Cerceris*). In addition, the phylogenetic signal we found in the models was always high (lambda > 0.79), and such high signals additionally make the use of close relatives less problematic. At last, our set of species could not determine what happened before the first appearance of highly mobile prey (e.g., in Mellinidae). Indeed, the ancestral prey of apoid wasps is believed to be a weakly‐ to non‐flying prey, probably cockroaches as in the basal family Ampulicidae (Bohart and Menke 1976). A larger set of species could shed further light on the evolution of visual system in the Apoidea.

## Author Contributions

**Chiara Francesca Trisoglio:** data curation (equal), formal analysis (equal), investigation (equal), methodology (equal), resources (equal), validation (equal), visualization (equal), writing – original draft (equal), writing – review and editing (equal). **Andrea Ferrari:** data curation (equal), formal analysis (equal), investigation (equal), methodology (equal), resources (equal), validation (equal), visualization (equal), writing – review and editing (equal). **Matteo Brilli:** data curation (equal), formal analysis (equal), investigation (equal), methodology (equal), validation (equal), visualization (equal), writing – review and editing (equal). **Michele Zilioli:** data curation (equal), investigation (equal), methodology (equal), resources (equal), writing – review and editing (equal). **Carlo Polidori:** conceptualization (lead), data curation (equal), formal analysis (equal), funding acquisition (lead), investigation (equal), methodology (equal), project administration (lead), resources (equal), supervision (lead), validation (equal), visualization (equal), writing – original draft (equal), writing – review and editing (equal).

## Funding

The study was supported by the project CGL2017‐83046‐P from Ministerio de Ciencia, Innovación y Universidades (Spain) to C.P. and by SYNTHESYS grant at Museo Nacional de Ciencias Naturales (CSIC) (ES‐TAF‐5333) to C.P.

## Conflicts of Interest

The authors declare no conflicts of interest.

## Supporting information


**Dataset S1:** Measurements taken on all the studied individuals, with origin of the sample, microscopy methodology, food item mobility and prey taxon. DNA sequences retrieved from GenBank used to reconstruct the phylogeny of the studied species are also provided.

**Table S1:** Full genomes used in the phylogenetic reconstruction.
**Table S2:** Transcriptome assemblies used in the phylogenetic reconstruction.
**Table S3:** Alignments and best model in the phylogenetic reconstruction.
**Table S4:** Simple linear regressions showing the relationships between head size and weighted size of visual traits. A significant regression (*p* < 0.05) indicates an allometric relationship. EyeA, eye area; HW, head width; OcD, ocellar diameter. In bold the *p*‐value for significant results.

## Data Availability

The complete dataset with the measurements taken on all specimens and details on the DNA sequences used to build the phylogeny are provided in Dataset S1.
