# Supplementary material for: Evolution of Peripheral Visual System in the Apoidea: A Role for Food Item Mobility?
Source: Ecol Evol. 2026 May 5;16(5):e73608. doi: 10.1002/ece3.73608 (PMC13143416; doi:10.1002/ece3.73608)
Supplement: Supplementary file 2 — Table S1: Full genomes used in the phylogenetic reconstruction. Table S2: Transcriptome assemblies used in the phylogenetic reconstruction. Table S3: Alignments and best model in the phylogenetic reconstruction. Table S4: Simple linear regressions showing the relationships between head size and weighted size of visual traits. A significant regression (p < 0.05) indicates an allometric relationship. EyeA, eye area; HW, head width; OcD, ocellar diameter. In bold the p‐value for significant results. [file ECE3-16-e73608-s002.docx]

Supporting information for the ms:

Evolution of peripheral visual system in the Apoidea: a role for food item mobility?

BY

Chiara Francesca Trisoglio, Andrea Ferrari, Matteo Brilli, Michele Zilioli, Carlo Polidori

**Table S1.** Full genomes used in the phylogenetic reconstruction.

| **Accession** | **Species** |
| --- | --- |
| GCA_028453745.1 | *Agapostemon virescens* |
| GCA_960531205.1 | *Andrena bicolor* |
| GCA_960531515.1 | *Andrena bicolor* |
| GCA_947577235.1 | *Andrena bucephala* |
| GCA_947577245.1 | *Andrena bucephala* |
| GCA_029168295.1 | *Andrena camellia* |
| GCA_029448645.1 | *Andrena camellia* |
| GCA_963855975.1 | *Andrena chrysosceles* |
| GCA_929108735.1 | *Andrena dorsata* |
| GCA_929114765.1 | *Andrena dorsata* |
| GCA_963971345.1 | *Andrena flavipes* |
| GCA_963971385.1 | *Andrena flavipes* |
| GCA_946251835.1 | *Andrena fulva* |
| GCA_946251845.1 | *Andrena fulva* |
| CAMITY01.1 | *Andrena fulva* |
| GCA_910592125.1 | *Andrena haemorrhoa* |
| GCA_910592295.1 | *Andrena haemorrhoa* |
| GCA_944738655.2 | *Andrena hattorfiana* |
| GCA_944738785.2 | *Andrena hattorfiana* |
| GCA_964234915.1 | *Andrena helvola* |
| GCA_964234935.1 | *Andrena helvola* |
| GCA_963932335.1 | *Andrena marginata* |
| GCA_963932365.1 | *Andrena marginata* |
| GCA_929113085.1 | *Andrena minutula* |
| GCA_929113495.1 | *Andrena minutula* |
| GCA_964214045.1 | *Andrena nitida* |
| GCA_964214085.1 | *Andrena nitida* |
| GCA_963932255.1 | *Andrena praecox* |
| GCA_963932275.1 | *Andrena praecox* |
| GCA_951212825.1 | *Andrena trimmerana* |
| GCA_951215215.1 | *Andrena trimmerana* |
| GCA_952773225.1 | *Andrena trimmerana* |
| GCA_022405125.1 | *Anthidium xuezhongi* |
| GCA_003314205.2 | *Apis mellifera mellifera* |
| GCA_028455555.1 | *Augochlorella aurata* |
| GCF_024516045.1 | *Bombus affinis* |
| GCA_037178635.1 | *Bombus dahlbomii* |
| GCA_041682495.2 | *Bombus fervidus* |
| GCA_911387925.2 | *Bombus hypnorum* |
| GCA_014825875.1 | *Bombus ignitus* |
| GCA_964186655.1 | *Bombus lapidarius* |
| GCA_905332965.1 | *Bombus pascuorum* |
| GCA_910591885.2 | *Bombus terrestris* |
| GCA_963989415.1 | *Cerceris ruficornis* |
| NW_017106536.1 | *Habropoda laboriosa* |
| GCA_028454255.1 | *Halictus ligatus* |
| GCA_028454245.1 | *Halictus quadricinctus* |
| GCA_028454235.1 | *Halictus rubicundus* |
| GCA_000346575.1 | *Lasioglossum albipes* |
| GCA_028454225.1 | *Lasioglossum leucozonium* |
| GCA_916610235.2 | *Lasioglossum morio* |
| GCA_046254535.1 | *Megachile sculpturalis* |
| GCA_945859595.2 | *Megachile willughbiella* |
| GCA_030673865.1 | *Melipona bicolor* |
| GCA_012274295.1 | *Osmia lignaria* |
| GCA_933228935.1 | *Pemphredon_lugubris* |
| GCA_030770465.2 | *Perdita meconis* |
| GCA_047676405.1 | *Perdita perpallida* |
| KY994149.1 | *Psenulus pallipes (mitochondrion)* |
| GCA_913789895.3 | *Seladonia tumulorum* |
| GCA_963989405.1 | *Trypoxylon_attenuatum* |
| GCA_963969225.2 | *Xylocopa violacea* |

**Table S2.** Transcriptome assemblies used in the phylogenetic reconstruction.

| **Accession** | **Species** |
| --- | --- |
| GBUA01.1 | *Alysson spinosus* |
| GHFR01.1 | *Andrena fulva* |
| GBLF01.1 | *Andrena vaga* |
| GBQR01.1 | *Bembix rostrata* |
| GBNS01.1 | *Cerceris arenaria* |
| GBOM01.1 | *Chalybion californicum* |
| GBWG01.1 | *Crabro peltarius* |
| GBWH01.1 | *Crossocerus quadrimaculatus* |
| KEJF01.1 | *Eucera angustior* |
| GBNR01.1 | *Gorytes laticinctus* |
| GBQK01.1 | *Heriades truncorum* |
| GBPY01.1 | *Isodontia mexicana* |
| GBTQ01.1 | *Lestica clypeata* |
| GBMJ01.1 | *Lithurgus chrysurus* |
| GBQU01.1 | *Nitela sp AD-2014* |
| KCAW01.1 | *Nomada maculata* |
| KFDC01.1 | *Nomada signata* |
| GBOC01.1 | *Palarius histrio* |
| GBQH01.1 | *Pemphredon lugens* |
| GBNH01.1 | *Psenulus fuscipennis* |
| GBNW01.1 | *Pseudoscolia sinaitica* |
| GAXN01.1 | *Sceliphron caementarium* |
| GBNB01.1 | *Stizus continuus* |

**Table S3.** Alignments and best model in the phylogenetic reconstruction.

| **ID** | **Type** | **Seq** | **Site** | **Unique** | **Infor** | **Invar** | **Const** | **Best model** |
| --- | --- | --- | --- | --- | --- | --- | --- | --- |
| 18S | DNA | 97 | 770 | 212 | 170 | 570 | 570 | TNe+I+G4 |
| 28S | DNA | 168 | 1190 | 623 | 232 | 723 | 722 | GTR+F+I+G4 |
| CAD | DNA | 101 | 1329 | 918 | 554 | 506 | 506 | TNe+G4 |
| COI | DNA | 218 | 664 | 551 | 423 | 187 | 187 | GTR+F+I+G4 |
| EF1a | DNA | 228 | 1228 | 779 | 519 | 660 | 660 | TIMe+I+G4 |
| LWRh | DNA | 202 | 660 | 498 | 349 | 260 | 260 | TIM2e+I+G4 |
| NaKATPase | DNA | 119 | 1466 | 690 | 585 | 835 | 835 | GTR+F+I+G4 |
| RNApol | DNA | 121 | 818 | 352 | 283 | 514 | 514 | TIM+F+I+G4 |
| wingless | DNA | 121 | 403 | 235 | 168 | 200 | 200 | TN+F+I+G4 |

**Table S4.** Simple linear regressions showing the relationships between head size and weighted size of visual traits. A significant regression (*P* < 0.05) indicates an allometric relationship. HW: head width, OcD: ocellar diameter, EyeA: eye area. In bold the *P*-value for significant results.

| **Group** | **Model** | ***R^2^*** | ***N*** | ***P*** |
| --- | --- | --- | --- | --- |
| *FLr*-species | HW vs. weighted OcD | 0.32 | 33 | **0.0005** |
|  | HW^2^ vs. weighted EyeA | 0.008 | 33 | 0.63 |
| *LMr*-species | HW vs. weighted OcD | 0.37 | 23 | **0.002** |
|  | HW^2^ vs. weighted EyeA | 0.03 | 23 | 0.44 |
| *HMr*-species | HW vs. weighted OcD | 0.03 | 17 | 0.49 |
|  | HW^2^ vs. weighted EyeA | 0.02 | 21 | 0.54 |
